# An integrated database of wood-formation related genes in plants

**DOI:** 10.1038/srep11422

**Published:** 2015-06-16

**Authors:** Ting Xu, Tao Ma, Quanjun Hu, Jianquan Liu

**Affiliations:** 1 State Key Laboratory of Grassland and Agro-Ecosystems, School of Life Sciences, Lanzhou University, Lanzhou 730000, Gansu, China

## Abstract

Wood, which consists mainly of plant cell walls, is an extremely important resource in daily lives. Genes whose products participate in the processes of cell wall and wood formation are therefore major subjects of plant science research. The Wood-Formation Related Genes database (WFRGdb, http://me.lzu.edu.cn/woodformation/) serves as a data resource center for genes involved in wood formation. To create this database, we collected plant genome data published in other online databases and predicted all cell wall and wood formation related genes using BLAST and HMMER. To date, 47 gene families and 33 transcription factors from 57 genomes (28 herbaceous, 22 woody and 7 non-vascular plants) have been covered and more than 122,000 genes have been checked and recorded. To provide easy access to these data, we have developed several search methods, which make it easy to download targeted genes or groups of genes free of charge in FASTA format. Sequence and phylogenetic analyses are also available online. WFRGdb brings together cell wall and wood formation related genes from all available plant genomes, and provides an integrative platform for gene inquiry, downloading and analysis. This database will therefore be extremely useful for those who focuses on cell wall and wood research.

Plant cells are encased by complex polysaccharide walls, which have diverse functions. These walls constitute the main component of wood, which has served as fuel for fires and been exploited for numerous other uses since human civilization began. Genetic analyses of the formation of plant cell walls have provided the basis for much of the current understanding of cell walls, including how walls are made, how their development is regulated, and how they function. At present, around 800 of the genes in the *Arabidopsis* genome are believed to be related to the formation of cell walls. Several databases based mainly on *Arabidopsis* genes have been constructed, including the Cell Wall Genomics database (http://cellwall.genomics.purdue.edu) and Cell Wall Navigator (http://cellwall.ucr.edu/Cellwall/), which brings together cell-wall related genes from *Arabidopsis*, rice and maize^1^,^2^,^3^. CAZy (http://www.cazy.org/) is another such database; it focuses on the genes encoding proteins that catalyze the synthesis of carbohydrates and glycoconjugates^4^. In recent years, numerous plant genomes, including those of some trees, have been published, but cell wall synthesis related genes are not covered in most of these genomes on those databases. In this study, we developed an integrated database of Wood-Formation Related Genes (WFRGs) from all plant species whose genomes are available. This database will provide a comprehensive and robust platform allowing researchers focusing on plant cells and wood to index, BLAST and determine the phylogenetic relationships of their genes of interest that are related to wood formation.

## Result

### Data organization

The plants with available genome sequences were classified into three groups on the basis of life history:

1. Herbaceous species with no obvious woody stems.
2. Woody species with woody stems.
3. Non-vascular plants, including mosses and algae.

In order to make the user interface more friendly, we abbreviated the species name in our database by using only the first letter of the genus to represent the genus (for example, Athaliana for *Arabidopsis thaliana*; see Table S1 for more details).

All genes were classified into 8 broad types according to function:

1. Cellulose and hemicellulose synthesis, comprising genes that encode proteins synthesizing cellulose and hemicellulose, the main components of plant cell walls.
2. Lignin synthesis, including genes that encode enzymes catalyzing the monolignol biosynthetic pathway and monolignol assembly.
3. Esterases, comprising genes that encode enzymes hydrolyzing esters, chemical compounds that contain a carbonyl group adjacent to an ether linkage.
4. Monosaccharide inter-conversion, including genes that encode enzymes catalyzing inter-conversions between nucleotide-diphospho-sugars (NDP-sugars, fundamental components of diverse polysaccharides and glycoconjugates).
5. Lyases, comprising genes that encode pectin/rhamnogalacturonan lyases.
6. Cell wall structural proteins, including genes that encode proteins playing important roles in plant cell wall structure.
7. Cell growth and other wood-formation related genes.
8. Transcription factors.

A total of 47 gene families/super families and 33 transcription factors were included in our database (see Table S2).

### Data access and utility of the database

Users gain access to the data in WFRGdb via a search. The Search function in our database is divided into two parts: BLAST Search and Main search.

The user interface for BLAST Search is similar to that at NCBI. After users have submitted their FASTA sequences or FASTA files, the database will return the results as a table and the result sequences are made available for download.

The Main search is made up of three parts: Information Search, Gene Search and Fast Search. Gene families, gene names (obtained by searching for a sequence via a gene name), related references and information about genomes can be accessed via Information Search. Gene Search and Fast Search return similar results. Gene Search has a more complex user interface allowing users to view details of gene families and genomes, while Fast Search has a relatively simple user interface and delivers results in a condensed format which is especially suitable for searching through a large amount of data.

To carry out an Information Search, users should choose an option and enter the term for which they want to search into the text box. For Gene Search and Fast Search, users should tick to select at least one gene family and one genome.

In the results of Information Search, the keywords are highlighted in red to make the results easier to read. The results of Gene Search and Fast Search are displayed in the form of a multifunctional table which supports paging and sorting. Clicking a gene name will open a detailed information box from which users can download the gene’s sequence. Similarly, when a gene family name is clicked, detailed information about the family will pop up in an information box.

To the left of the gene name is a row of checkboxes; users can check these to download all selected gene sequences in a FASTA file or use all the checked gene sequences for further analysis.

**Figure 1:**
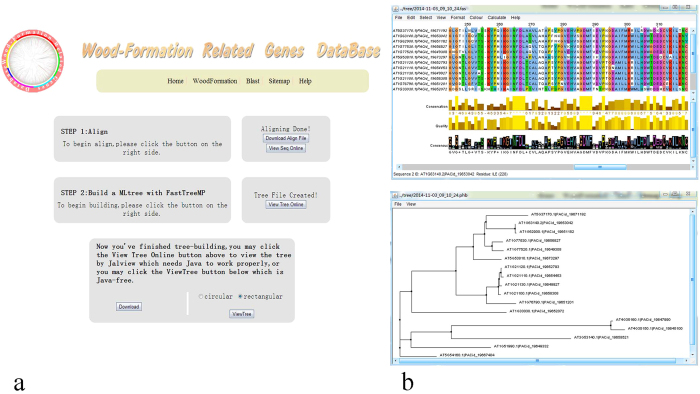
Data analysis page from WFRGdb. The user interface for analysis work is shown in Fig. 1a. Figure 1b shows the sequence analysis window (top) and tree analysis page (bottom).

**Figure 2:**
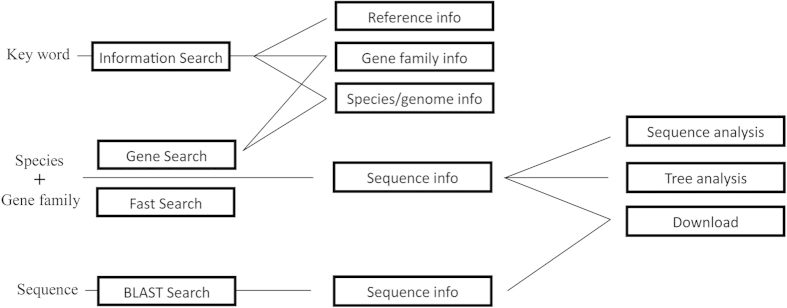
A schematic overview of WFRGdb. Information Search requires key words as input, while Gene Search and Fast Search need Species and Gene family as input information and BLAST Search needs sequence data.

The “Go Analysis” button on the bottom right of the page will take users to the analysis page. By following the instructions on this page, users can complete sequence analyses step by step. Both sequence and phylogenetic analyses are available on this page and all related files can also be downloaded. Alignment is done by Clustal W and maximum likelihood trees are built by FastTree^5^,^6^. Sequence analysis and tree analysis are implemented in JalView, which requires Java support (see Fig. 1)^7^. Users therefore need to install Java on their computers in advance. Our database also provides a simple function called jsPhyloSVG, which is Java-independent, for viewing tree files online^8^. As it may take some time to finish sequence analyses, we provide an e-mail service. If an e-mail address is supplied, the results (including gene sequences in FASTA format, aligned sequence files and tree files in Newick format) will be sent to users once the work has been done in the background. A schematic overview of information flow in WFRGdb is shown in Fig. 2.

## Discussion

WFRGdb uses all plant genomes whose sequences have been released to date to search out all known genes involved in wood formation. It is designed to assist researchers in finding and identifying all genes orthologous to their targets related to plant cell wall and wood formation, and in constructing their phylogenetic relationships. In the case of a gene family, researchers can obtain all sequences that belong to this family, and a phylogenetic tree for the family is also available. General information about the family is also easily accessible with the help of the references recommended in the database. We believe that WFRGdb will be very useful for those focusing on cell wall and wood research.

To our knowledge, WFRGdb is the first comprehensive resource database related to cell-wall and wood-formation related genes based on the mass of genome data now available. Here we present a collection of putative genes involved in wood-formation and display them in the form of gene families/super families and according to species/genomes, as well as providing easy access for data downloading and sequence analysis.

## Method

### Data sources

**Figure 3:**
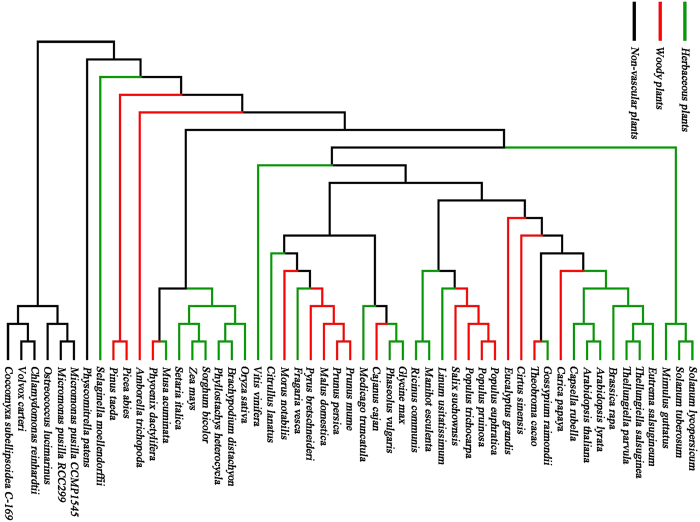
Phylogenetic tree showing all the species recorded in WFRGdb. Herbaceous plants are indicated in green and woody plants are in red. For some species (e.g. *Gossypium raimondii*), two or more genome sequences have been published; in such cases, all available sequences are covered in WFRGdb (see Table S1).

To date, 57 genomes for 28 herbs, 22 trees and 7 non-vascular species have been published (Fig. 3 and Table S1)^9^,^10^,^11^,^12^,^13^,^14^,^15^,^16^,^17^,^18^,^19^,^20^,^21^,^22^,^23^,^24^,^25^,^26^,^27^,^28^,^29^,^30^,^31^,^32^,^33^,^34^,^35^,^36^,^37^,^38^,^39^,^40^,^41^,^42^,^43^,^44^,^45^,^46^,^47^,^48^,^49^,^50^,^51^,^52^,^53^,^54^,^55^,^56^,^57^,^58^,^59^,^60^,^61^,^62^,^63^. Genome data were obtained mainly from Phytozome (http://www.phytozome.net, a joint project of the Department of Energy’s Joint Genome Institute and the Center for integrative Genomics to facilitate comparative genomic studies amongst green plants) or from dedicated genome websites for individual targeted species.

### Gene prediction

After downloading the genome data, for each gene where the GFF file indicated the existence of alternatively spliced transcripts, we discarded all but the longest such transcript. The proteins encoded by all the downloaded gene sequences were entered into a BLAST protein database^64^. We collected the sequences of all known Arabidopsis members of 47 gene families related to cell wall and wood synthesis from the internet, 33 transcription factors from Plant Transcription Factor Database(PTFB)^65^, and used them as the initial query in a BLAST search against our protein database. All hits obtained in this search were flagged as candidate genes. We examined each of these candidate genes in order to ensure that it belonged to the ascribed family. To do this, we ran each of these candidate protein sequences against the protein database again and examined the top 10 non-self hits for each gene in the resulting list. A candidate gene was removed if two or more of the top 10 non-self hits were not members of the 47 gene families.

The candidates retained after this analysis were then tested further using HMMER to ensure that each shared the domain /domains of the gene family to which it belonged^66^. The domain information for the gene families was derived from PFAM (http://pfam.sanger.ac.uk), a database of protein families that are represented by multiple sequences generated using hidden Markov models. The candidates that passed the HMMER tests were retained. Finally, the coding DNA sequence (CDS) of each gene was extracted from the CDS section of its GFF file by an in-house Perl script.

## Additional Information

**How to cite this article**: Xu, T. *et al.* An integrated database of wood-formation related genes in plants. *Sci. Rep.* **5**, 11422; doi: 10.1038/srep11422 (2015).

## Supplementary Material

Supplementary Information
