# Supplementary material for: An integrated database of wood-formation related genes in plants
Source: Sci Rep. 2015 Jun 16;5:11422. doi: 10.1038/srep11422 (PMC4468578; doi:10.1038/srep11422)
Supplement: Supplementary Information [file srep11422-s1.pdf]

Supplemental materials

An integrated database of wood-formation related genes in plants

Ting Xu#, Tao Ma#, Quanjun Hu, Jianquan Liu*

State Key Laboratory of Grassland and Agro-Ecosystems, School of Life Sciences, Lanzhou University, Lanzhou 730000, Gansu, China

*Corresponding author: Dr Jianquan Liu, liujq@lzu.edu.cn

State Key Laboratory of Grassland and Agro-Ecosystems, School of Life Sciences, Lanzhou University, Lanzhou 730000, Gansu, China

Supplemental tables

Supplemental Table 1. Source information of genomes collected in WFRGdb.

Latin name	Name	Name in WFRGdb	Data source
Amborella trichopoda	Amborella	Atrichopoda	http://www.amborella.org/
Arabidopsis lyrata	Lyrate rockcress	Alyrata	Phytozome_v9.0
Arabidopsis thaliana	Thale cress	Athaliana	http://www.arabidopsis.org/
Brachypodium distachyon	Purple false brome	Bdistachyon	http://www.brachypodium.org/
Brassica rapa	Chinese cabbage	Brapa	http://brassicadb.org/brad
Cajanus cajan	Pigeon pea	Ccajan	http://cajca.comparative-legumes.org/
Capsella rubella	Red Shepherd's Purse	Crubella	Phytozome_v9.0
Carica papaya	Papaya	Cpapaya	http://asgpb.mhpcc.hawaii.edu/papaya
Chlamydomonas reinhardtii	Chlamydomonas reinhardtii	Creinhardtii	Phytozome_v9.0
Citrullus lanatus	Watermelon	Clanatus	http://www.iwgi.org/
Citrus sinensis (2012NG)	Sweet orange	Csinensis(2012NG)	http://citrus.hzau.edu.cn/orange/index.php
Citrus sinensis (SOGP2010)	Sweet orange	Csinensis(SOGP2010)	http://www.citrusgenomedb.org/species/sinensis
Coccomyxa subellipsoidea C-169	Coccomyxa subellipsoidea	Csubellipsoidea C-169	Phytozome_v9.0
Eucalyptus grandis	Rose gum	Egrandis	Phytozome_v9.0
Eutrema halophilum	Eutrema halophilum	Ehalophilum	Phytozome_v9.0
Fragaria vesca	Woodland strawberry	Fvesca	http://www.rosaceae.org/species/fragaria/fragaria_vesca/
Glycine max	Soybean	Gmax	Phytozome_v9.0
Gossypium raimondii (2012Nature)	Cotton	Graimondii(2012Nature)	Phytozome_v9.0
Gossypium raimondii (2012NG)	Cotton	Graimondii(2012NG)	http://cgp.genomics.org.cn/page/species/index.jsp
Linum usitatissimum	Flax	Lusitatissimum	Phytozome_v9.0
Malus domestica	Apple	Mdomestica	Phytozome_v9.0
Manihot esculenta	Cassava	Mesculenta	Phytozome_v9.0
Medicago truncatula	Barrel Medic	Mtruncatula	http://medicago.org/genome/
Micromonas pusilla CCMP1545	Micromonas pusilla	Mpusilla CCMP1545	Phytozome_v9.0
Micromonas pusilla RCC299	Micromonas pusilla	Mpusilla RCC299	Phytozome_v9.0
Mimulus guttatus	Monkey-flower	Mguttatus	Phytozome_v9.0
Morus notabilis	Morus notabilis	Mnotabilis	http://morus.swu.edu.cn/morusdb/
Musa acuminata	Banana	Macuminata	http://banana-genome.cirad.fr/
Oryza sativa	Asian rice	Osativa	Phytozome_v9.0
Ostreococcus lucimarinus	Ostreococcus lucimarinus	Olucimarinus	Phytozome_v9.0
Phaseolus vulgaris	Common bean	Pvulgaris	Phytozome_v9.0
Phoenix dactylifera	Date palm	Pdactylifera	http://qatar-weill.cornell.edu/research/datepalm/index.html
Phyllostachys heterocycla	Moso bamboo	Pheterocycla	http://www.ncgr.ac.cn/bamboo
Physcomitrella patens	Physcomitrella patens	Ppatens	Phytozome_v9.0
Picea abies	Norway spruce	Pabies	http://congenie.org/
Pinus taeda	Loblolly pine	Ptaeda	http://dendrome.ucdavis.edu/treegenes/
Populus euphratica	Euphrates poplar	Peuphratica	-
Populus pruinosa	Populus pruinosa	Ppruinosa	-
Populus trichocarpa	Western Poplar	Ptrichocarpa	Phytozome_v9.0
Prunus mume	Mei	Pmume	http://prunusmumegenome.bjfu.edu.cn/
Prunus persica	Peach	Ppersica	Phytozome_v9.0
Pyrus bretschneideri	Chinese white pear	Pbretschneideri	http://peargenome.njau.edu.cn/
Ricinus communis	Castor	Rcommunis	http://castorbean.jcvi.org/index.php
Salix suchowensis	Salix integra	Ssuchowensis	-
Selaginella moellendorffii	Spikemoss	Smoellendorffii	Phytozome_v9.0
Setaria italica (2012NBt_China)	Foxtail millet	Sitalica(2012NBt_China)	ftp://ftp.genomics.org.cn/pub/Foxtail_millet/
Setaria italica (2012NBt_USA)	Foxtail millet	Sitalica(2012NBt_USA)	Phytozome_v9.0

Latin name	Name	Name in WFRGdb	Data source
Solanum lycopersicum	Tomato	Slycopersicum	http://solgenomics.net/organism/Solanum_tuberosum/genome
Solanum tuberosum	Potato	Stuberosum	http://solanaceae.plantbiology.msu.edu/pgsc_download.shtml
Sorghum bicolor	Sorghum	Sbicolor	Phytozome_v9.0
Thellungiella salsuginea	Salt cress	Tsalsuginea	http://thellungiella.org/
Thellungiella parvula	Thellungiella parvula	Tparvula	http://thellungiella.org/
Theobroma cacao (2011NG)	Cacao tree	Tcacao(2011NG)	http://cocoagendb.cirad.fr/
Theobroma cacao (2013GB)	Cacao tree	Tcacao(2013GB)	Phytozome_v9.0
Vitis vinifera	Grape vine	Vvinifera	http://www.genoscope.cns.fr/spip/Vitis-vinifera-e.html
Volvox carteri	Volvox carteri	Vcarteri	Phytozome_v9.0
Zea mays	Maize	Zmays	http://www.maizegdb.org/

Supplemental Table 2. Gene families covered in WFRGdb.

Classification in WFRGdb	Gene family	Description
Cellulose and Hemicellulose synthesis	CSL	Cellulose synthases，Cellulose synthase-like Genes，Hemicelulose biosynthesis
	GSL	Callose Synthase,Callose Synthase-like genes,involved in synthesis of the cell-wall component callose in specialized locations
	RGP	Reversibly glycosylated polypeptides
	XFT	Xyloglucan Fucosyltransferase,GT37
	XGT	Xyloglucan galactosyltransferase,GT47
	XXT	Xyloglucan xylosyltransferases and galactomannan galactosyltransferases,GT34
	GH18	glycoside hydrolase 18 ,chitinases
	GH1	glycoside hydrolase 1,involved in chemical defense against herbivory, lignification, hydrolysis of cell wall-derivedoligos acchari des during germination
	GH10	glycoside hydrolase 10,formerly known as cellulase family F
	GH16	glycoside hydrolase 1,involved in Reorganization and degradation of the wall crosslinking and seed storage polysaccharide
	GH17	glycoside hydrolase 17
	GH27	glycoside hydrolase 27
	GH28	glycoside hydrolase 28
	GH3	glycoside hydrolase 3
	GH31	glycoside hydrolase 31
	GH35	glycoside hydrolase 35
	GH43	glycoside hydrolase 43 AXH/arabinoxylan arabinofuranohydrolase
	GH5	glycoside hydrolase 5
	GH51	glycoside hydrolase 51 arabinofuranosidase hydrolyze related
	GH79	glycoside hydrolase 79
	GH9	glycoside hydrolase 9 ,cellulases
	GT8	GlycosylTransferase 8
Lignin synthesis	4CL	4-coumarate:CoA ligase，lignin biosynthesis related,a key enzyme of phenylpropanoid metabolism
	C3HC4HF5H	lignin biosynthesis related,p-Coumarate 3-hydroxylase (C3H),Trans-cinnamate 4-hydroxylase (C4H),Ferulate 5-hydroxylase (F5H),lignin biosynthesis related
	CAD	cinnamyl alcohol dehydrogenase gene，lignin biosynthesis related,a key enzyme catalyzes the final step in the synthesis of monolignols.
	CCR	Cinnamoyl CoA reductase，lignin biosynthesis related,catalyzes the first step of the phenylpropanoid pathway specifically dedicated to the monolignol biosynthetic branch
	CCoAOMT	caffeoyl-coenzyme A 3-O-methyltransferase,lignin biosynthesis related,mediates the methylation of hydroxylated monomeric lignin
	COMT	Caffeic acid O-methyltransferase,lignin biosynthesis related
	HCT	hydroxycinnamoyltransferase ,lignin biosynthesis related.
	Laccase	Laccase,play a role in the formation of lignin by promoting the oxidative coupling of monolignols
	PAL	Phe ammonia lyase,lignin biosynthesis related, catalyses the first step of the phenylpropanoid pathway
Esterase	PAE	Pectin Acetylesterase Gene,catalyzes the deacetylation of pectin
	PMEI	Pectin methylesterases,catalyse the demethylesterification of cell wallpolygalacturonans
	PMEII	Pectin methylesterases,catalyse the demethylesterification of cell wallpolygalacturonans
	FE	feruloyl esterases,CE1
Monosaccharide Inter-conversion	GMP	GDP-mannose pyrophosphorylase
	NSE	Nucleotide-Sugar Interconversion Enzymes,interconverts one NDP-sugar to another
	UGDH	Uridine diphosphate (UDP)-glucose dehydrogenase,s a key enzyme required for synthesis of glycosaminoglycans (GAGs) and for detoxification of toxins, drugs, and endogenous substances
Monosaccharide Inter-conversion	UGP	UDP-glucose pyrophosphorylase,catalyzing a reversible production of UDPG and pyrophosphate (PPi) from Glc-1-P and UTP

Classification in WFRGdb	Gene family	Description
Lyases	PL1	Pectate and pectin lyase-like genes
	RGIL	rhamnogalacturonan lyases
Cell wall structure protein	AGP	arabinogalactan-protein encoding related； Cellwall structure protein
	GRP	glycine-rich structural protein,Cellwall structure protein
	HRGP	hydroxyproline-rich glycoprotein,Cellwall structure protein,important structuralcomponents of plantcellwalls,also accumulate in response to infection as an apparent defense mechanism
	LRRP	Leucine-Rich Repeat proteins,Cellwall structure protein
	PRP	proline-rich proteins,cellwall structure protein
Cell growth Related	Expansin	expansin,cell-wall-loosening proteins that induce stress relaxation and extension of plant cell walls
Transcription Factor	ABI3-VP1	a multidomain transcription factor that functions as both an activator and a repressor depending on the promoter context;act as intermediaries in regulating abscisic acid (ABA)－responsive genes during seed development
	Alfin-like	Alfin1 cDNA,obtained by differential screening of a poly(A)+ library from salt-tolerant alfalfa cells, encodes a novel protein with a Cys4 and His/Cys3 putative zinc-binding domain that suggests a possible role for this protein in transcriptional regulation
	AP2-EREBP	AP2-EREBP genes form a large multigene family,and they play a variety of roles throughout the plant life cycle:from being key regulators of several developmental processes,like floral organ identity determination or control of leaf epidermal cell identity,to forming part of the mechanisms used by plants to respond to various types of biotic and environmental stress
	ARF	a family of transcription factors that bind with specificity to auxin response elements (AuxREs) in promoters of primary or early auxin-responsive genes
	ARID	ARID-encoding genes are involved in a variety of biological processes including embryonic development, cell lineage gene regulation and cell cycle control
	AUX-IAA	Aux-IAA proteins are short-lived nuclear proteins that repress expression of primary/early auxin response genes in protoplast transfection assays
	bHLH	function in lsquoplant-specificrsquo or lsquoanimal-specificrsquo processes.In animals bHLH proteins are involved in regulation of a wide variety of essential developmental processes.In Plants,this family of TFs has a range of different roles in plant cell and tissue development as well as plant metabolism.
	bZIP	In plants,basic region/leucine zipper motif (bZIP) transcription factors regulate processes including pathogen defence, light and stress signalling, seed maturation and flower development
	C2C2-CO-like	Phylogenetic analysis of the COL family demonstrated that it is organized into a few distinct groups, some of which evolved before the divergence of gymnosperms and angiosperms. Molecular evolutionary analyses showed that COL genes within the Brassicaceae family evolve rapidly.
	C2C2-Dof	plant-specific,function as a transcriptional activator or a repressor involved in diverse plant-specific biological processes
	C2C2-GATA	Members of this group have been identified in organisms ranging from cellular slime mold to vertebrates, including plants, fungi, nematodes, insects, and echinoderms.
	C2H2	C2H2 proteins belong to a group of transcription factors (TFs) existing as a superfamily that plays important roles in defense responses and various other physiological processes in plants
	C3H	AetTZF1 increased stress tolerance to drought by promoting root growth and increasing germination rates in Arabidopsis. Overexpression of AetTZF1led to an altered expression level of stress-related genes that made the plants more tolerant to drought.
	CAMTA	CAMTAs comprise a conserved family of transcription factors in a wide range of multicellular eukaryotes, which possibly respond to calcium signaling by direct binding of calmodulin
	CCAAT	Transcription factors belonging to the CCAAT-box binding factor family (also known as the Nuclear Factor Y) are present in all higher eukaryotes. Studies in plants have revealed that each subunit of this heterotrimeric transcription factor is encoded by a gene belonging to a multigene family allowing a considerable modularity

Classification in WFRGdb	Gene family	Description
Transcription Factor	CPP	CPP1 might be involved in the regulation of the leghemoglobin genes in the symbiotic root nodule
	E2F-DP	E2F-DP transcription factors are key components of the cyclin D/retinoblastoma/E2F pathway, they regulate the expression of genes required for G1/S transition and S-phase progression. Arabidopsis contains a family of functionally distinct E2F genes, most probably involved in the G1-to-S phase progression
	EIL	ETHYLENE-INSENSITIVE3 (EIN3) is a transcription factor that functions downstream from the ethylene receptors in the Arabidopsis ethylene signal transduction pathway.These transcription factors have been proposed to be functionally redundant positive regulators of multiple ethylene responses
	GeBP	GeBP is acting as a repressor of leaf cell fate
	GRAS	Members of the GRAS gene family encode transcriptional regulators that have diverse functions in plant growth and development such as gibberellin signal transduction, root radial patterning, axillary meristem formation, phytochrome A signal transduction, and gametogenesis
	GRF	GRF encodes a putative transcription factor that appears to play a regulatory role in stem elongation
	HB	The homeodomain is a DNA-binding motif within transcription factor proteins. These transcription factors may be involved in cell differentiation and control of cell growth, as well as patterning of diverse organisms
	HSF	HSF is present in a latent state under normal conditions; it is activated upon heat stress by induction of trimerization and high-affinity binding to DNA and by exposure of domains for transcriptional activity
	MADS	the human serum-response factor (SRF) is involved in co-ordinating transcription of the protooncogene c-fos, whilst MCM1 is central to the transcriptional control of cell-type specific genes and the pheromone response in the yeast Saccharomyces cerevisiae. The RSRF/MEF2 proteins comprise a sub-family of this class of transcription factors which are key components in muscle-specific gene regulation. Moreover, in plants, MADS-box proteins such as AG, DEFA and GLO play fundamental roles during flower development
	MYB	MYB proteins constitute a diverse class of DNA-binding proteins of particular importance in transcriptional regulation in plants. Members are characterised by having a structurally conserved DNA-binding domain, the MYB domain. Different categories of MYB proteins can be identified depending on the number of imperfect repeats of the MYB domain they contain.Functions of MYB proteins in plants include regulation of secondary metabolism, control of cellular morphogenesis and regulation of meristem formation and the cell cycle
	MYB-related	MYB proteins constitute a diverse class of DNA-binding proteins of particular importance in transcriptional regulation in plants. Members are characterised by having a structurally conserved DNA-binding domain, the MYB domain. Different categories of MYB proteins can be identified depending on the number of imperfect repeats of the MYB domain they contain.Functions of MYB proteins in plants include regulation of secondary metabolism, control of cellular morphogenesis and regulation of meristem formation and the cell cycle
	NAC	NAC (NAM, ATAF1/2 and CUC2) domain proteins are plant-specific transcriptional factors known to play diverse roles in various plant developmental processes. NAC transcription factors comprise of a large gene family represented by more than 100 members in Arabidopsis, rice and soybean etc.
	PHD	PHD proteins seem to be found universally in the nucleus, and their functions tend to lie in the control of chromatin or transcription. Increasing evidence indicates that PHD fingers bind to specific nuclear protein partners, for which they apparently use their loop 2 surface.
	SBP	The Arabidopsis thaliana SPL gene family represents a group of structurally diverse genes encoding putative transcription factors found apparently only in plants. The distinguishing characteristic of the SPL gene family is the SBP-box encoding a conserved protein domain of 76 amino acids in length, the SBP-domain, which is responsible for the interaction with DNA.

Classification in WFRGdb	Gene family	Description
Transcription Factor	TCP	The TCP domain is a plant-specific DNA binding domain found in proteins from a diverse array of species;involves in leaf development via the jasmonate signaling pathway.
	Trihelix	To date, DNA-binding proteins characterized by the trihelix motif have been described only in plants, and may therefore be involved in plant-specific processes.
	WRKY	WRKY transcription factors are one of the largest families of transcriptional regulators in plants and form integral parts of signalling webs that modulate many plant processes.New findings illustrate that WRKY proteins often act as repressors as well as activators, and that members of the family play roles in both the repression and de-repression of important plant processes. Furthermore, it is becoming clear that a single WRKY transcription factor might be involved in regulating several seemingly disparate processes. Mechanisms of signalling and transcriptional regulation are being dissected, uncovering WRKY protein functions via interactions with a diverse array of protein partners, including MAP kinases, MAP kinase kinases, 14-3-3 proteins, calmodulin, histone deacetylases, resistance proteins and other WRKY transcription factors.
	ZF-HD	ZF-HD class of homeodomain proteins may be involved in the establishment of the characteristic expression pattern of the C4 PEPCase gene.
